# In Situ Observation the Effect of Y on the Solidification Process of 7Mo-SASS under a Low Cooling Rate

**DOI:** 10.3390/ma16216846

**Published:** 2023-10-25

**Authors:** Wenqiang Liu, Lijun Wang, Qi Wang

**Affiliations:** 1 The Collaborative Innovation Center of Steel Technology, University of Science and Technology Beijing, Beijing 100083, China; d202110616@xs.ustb.edu.cn; 2 The Hebei Key Laboratory of Material Near-Net Forming Technology, School of Materials Science and Engineering, Hebei University of Science and Technology, Shijiazhuang 050018, China

**Keywords:** 7Mo austenite stainless steel, solidification process, confocal laser scanning microscope, low cooling rate, Y, JMAK, refinement

## Abstract

The effects of Y on the solidification process of 7Mo super austenitic stainless steel (7MoSASS) under low cooling rate conditions (10 °C/min) were investigated using high-temperature confocal laser scanning microscopy (HT-CLSM). The in situ observation results indicate that Y samples promote an increase in austenite nucleation density. After 10 s of nucleation, the nucleation density increased by 149.53/mm^2^ for the Y sample. Furthermore, variance analysis indicated that Y addition improved the uniformity of the 7MoSASS solidification microstructure under low cooling rate conditions. The Johnson–Mehl–Avrami–Kolmogorov (JMAK) theory results showed that when the solid phase ratio was 0.5, the nucleation mode of the Y sample transitioned from saturation site nucleation to saturation site nucleation + Avrami nucleation. YAlO_3_ has a low lattice disregistry value with austenite, making it a suitable heterogeneous nucleation core for promoting the early nucleation of austenite. During the late stages of solidification, Y accumulates in the residual liquid phase, providing a greater degree of compositional undercooling. SEM-EDS analysis showed that Y contributed to the refinement of the 7MoSASS solidification microstructure, with the proportion of precipitated phases decreasing by approximately 7.5%. Cr and Mo were the main elements exhibiting positive segregation in 7MoSASS, and the Cr segregation ratio increased in the Y sample, while the Mo segregation ratio decreased.

## 1. Introduction

In recent years, the high-end equipment manufacturing industry has developed rapidly, and the large scale, integration, and high performance of equipment is the development trend in the future. Super austenitic stainless steels (SASSs) are one of the key materials in high-end equipment manufacturing and exhibit excellent corrosion resistance and outstanding mechanical properties [1,2]. They have a wide application prospect in flue gas desulfurization, seawater desalination, and other fields [3,4,5]. In this process, the researchers overcame the difficulties of low Mo super austenitic stainless steel, such as 904L and S31254, and completed their own production. However, 7Mo-SASS, the highest grade of super austenitic stainless steel, is still in the experimental research stage [6,7,8]. The core problem is how to improve the issue of element segregation of high alloy content, such as Cr and Mo [9,10,11]. Therefore, it is important to study the solidification process of 7MoSASS and explore effective methods to improve the solidification microstructure and reduce element segregation.

Refining grain is commonly employed to improve the solidification microstructure. Physical methods, such as electromagnetic stirring and ultrasonic treatment, which require complex and costly equipment, have been traditionally used [12,13]. However, the addition of rare earth elements to achieve grain refinement has emerged as a research hotspot [14]. Rare earth elements have been widely used as microalloying elements in modifying inclusions and deeply purifying molten steel in low-alloy steels [15,16,17,18,19]. Researchers have shown great interest in incorporating rare earth elements into steel production. This approach offers dual advantages. Firstly, high melting point rare earth inclusions (such as RExOy and RExOySz) act as nucleation sites, promoting heterogeneous nucleation and effectively refining the solidification microstructure. Secondly, rare earth microalloying contributes to significant improvements in material properties, including enhanced corrosion resistance [20,21,22] and superior mechanical performance [23,24]. Currently, the application of rare earth elements in SASSs has become a focus [25,26,27,28,29]. Wang [27,29] and Zhang [25] have confirmed that Ce plays a role in grain size refinement and can improve the segregation of second phases in SASSs. However, there are few studies on the application of Y in SASSs. Mao et al. [30] investigated the modification mechanism of Y in hypoeutectic Al-Si alloys under different cooling rates. The results revealed that at low cooling rates, the addition of Y can ameliorate compositional undercooling and promote the nucleation of eutectic Si. Li et al. [31] investigated the influence of Y on the microstructure and eutectic solidification behavior of an Al-7.5%Si-0.45%Mg alloy. The addition of Y resulted in a decrease in the nucleation and growth temperature of the eutectic phase.

High-temperature confocal laser scanning microscope (HT-CLSM) can observe in situ, continuously, dynamically, and directly the changes in microstructure and phase during the melting and solidification of materials at high temperatures. HT-CLSM was employed to investigate the influence of cooling rate on SASSs. The results showed that as the cooling rate decreases, the segregation of Mo elements intensifies [32,33]. Many researchers have noticed that the addition of rare earths changes the crystallization temperature range and crystallization rate of steel [16,29,34]. Furthermore, in the actual solidification process of SASSs, the most pronounced occurrence of ingot segregation and grain coarsening take place in the central region. This phenomenon is attributed to the gradual reduction in cooling rate from the edges toward the center [32,35]. The influence of rare earth elements on the solidification process of 7MoSASS under low cooling speeds remains ambiguous.

In this work, the effect of Y on the solidification process at a low cooling rate (10 °C/min) was investigated through in situ observations, revealing its effects on the solidification process and segregation behavior of 7Mo-SASS. Specifically, we discussed the impact of Y on the solidification process of 7MoSASS at a low cooling rate, as well as its influence on the nucleation and growth of austenite grains during this process, providing a theoretical foundation for addressing the central segregation issue in billets.

## 2. Materials and Methods

### 2.1. Rare Earth Addition Experiment

**Figure 1:**
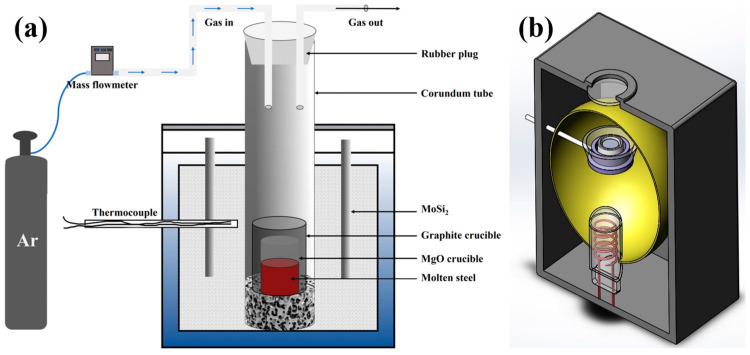
(**a**) Schematic diagram of the shaft furnace; (**b**) schematic diagram of the high-temperature confocal laser-scanning microscope.

The experimental 7MoSASS was obtained from TISCO (Taiyuan, China), and its chemical composition is shown in Table 1. The experiment of adding rare earth to 7MoSASS was completed by a vertical furnace. A 7MoSASS (450 g) sample was placed in a MgO crucible (Φ*L*50) and MoSi_2_ resistance furnace (Φ*L*90), as shown in Figure 1a. In a high-purity argon atmosphere, the temperature was raised to 1500 °C to achieve the complete melting of the 7MoSASS. To prevent oxidation, the rare earth metal was enveloped with reduced iron powder before being introduced into the liquid steel. Following this, the mixture was incorporated into the molten steel and stirred with a quartz tube for 15 s. Additionally, it was held at a steady temperature for 30 min to ensure the uniform distribution of rare earth elements. As shown in Table 1, the content of Y was obtained by inductive coupled plasma (ICP) analysis, while the content of other elements was obtained through a direct-reading spectrometer.

### 2.2. High-Temperature Confocal Laser Scanning Microscope Experiment

The in situ observation of the solidification process of 7MoSASS was performed using CLSM (Figure 1b) [2]. Samples were taken from the center of the experimental ingot and processed into disks (diameter 7.6 mm, height 2.5 mm) using wire electrical discharge machining. The samples were then polished to a mirror finish and placed in alumina crucibles, which were then placed in the chamber of a confocal laser scanning microscope (VL2000DX-SVF17SP, LASERTEC Inc., Yokohama, Japan). The sample chamber was evacuated multiple times using a vacuum pump and purified with ultra-pure argon gas to prevent surface oxidation of the samples. The samples were heated with a heating rate of 200 °C/min to 1300 °C, followed by a heating rate of 50 °C/min to 1450 °C, and held isothermally for 5 min. The cooling rate was set to 10 °C/min to study the solidification behavior of all samples. Previously, Zhang et al. [36] found that the equipment (the same used in this experiment) displayed a temperature difference of approximately 50 °C between the set temperature and the surface temperature.

To observe the inclusions in the steel, the steel sample was ground to 2000 grit using SiC abrasive papers and then polished with diamond paste. The chemical compositions and morphologies of inclusions were analyzed through scanning electron microscopy and energy dispersive spectroscopy (SEM-EDS, Zeiss EVO18, Zeiss, Jena, Germany). Each sample was observed with 10 fields of view under magnification 1000 times.

## 3. Results and Discussion

### 3.1. Effect of Y on the Solidification Process of 7Mo SASS

**Figure 2:**
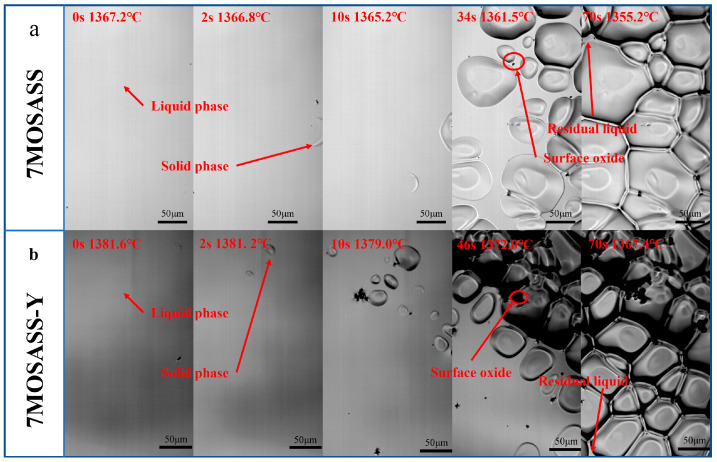
Solidification process of 7MoSASS observed in situ via HT-CLSM and cooled at 10 °C/min: (**a**) 7MOSASS; (**b**) 7MOSASS-Y.

Figure 2 illustrates the solidification process of 7MoSASS and 7MoSASS-Y at a low cooling rate of 10 °C/min, showing the initial nucleation, nucleation after 2 s, nucleation after 10 s, solid fraction of 50%, and solid fraction of 99%, with time and temperature labels. In situ observations reveal that the actual liquidus temperature of 7MoSASS is 1367.2 °C. This discrepancy is primarily attributed to the cooling rate and solidification conditions. During solidification, the initial grains of 7MoSASS take on a crescent-shaped morphology as they precipitate from the liquid phase. Early-formed grains have a growth advantage, reaching sizes exceeding 100 μm. As the system temperature gradually decreases, the undercooling of the steel increases, promoting the nucleation of austenite and subsequently completing the solidification process. The addition of Y significantly influences the solidification process of 7MoSASS. The Y sample exhibits ellipsoidal initial grains. By considering the data in Table 2, it can be observed that the nucleation temperature is advanced by 14.4 °C for the Y sample. Furthermore, the grain density growth rates (v = (g(10) − g(2))/8, where g(10) and g(2) represent the grain density at 2 s and 10 s, respectively, for the Y sample are substantially higher than 7MoSASS, with increases of 15.46 mm^−2^ s^−1^. Therefore, Y can effectively promote the nucleation process of 7MoSASS during solidification.

**Figure 3:**
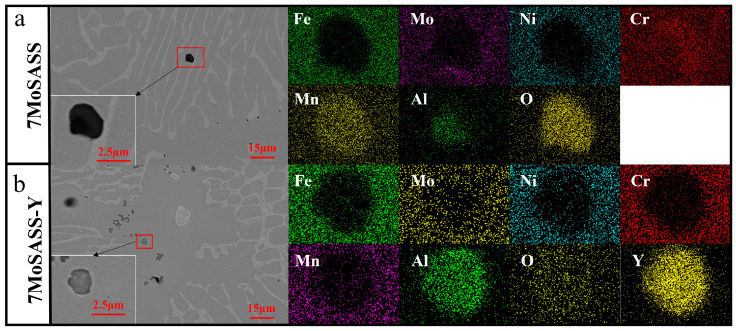
SEM photos of inclusions in CLSM samples. (**a**) 7MoSASS, (**b**) 7MoSASS-Y.

The statistical analysis of grain size within the field of view yields the following observations, as presented in Table 3 and Figure 3. Notably, 7MoSASS exhibits the highest range (greatest variation) in grain size, indicating a higher level of heterogeneity in grain sizes for 7MoSASS. Further, variance analysis was conducted to assess the impact of Y on grain size uniformity. Analysis of variance (α) is a statistical method used to examine differences in a continuous outcome variable based on different categories or groups defined by discrete factors [37]. The results reveal that 7MoSASS-Y exhibits the smallest variance in grain size. Taking into account the variance results, it can be inferred that at a low cooling rate (10 °C/min), the addition of Y is more effective in improving the solidification microstructure of 7MoSASS.

### 3.2. Effect of Y Addition on the Nucleation Process in 7MoSASS

Under the composition of the experimental steel, the predominant inclusions in the steel were identified as M_2_O_3_ (M = Al, Cr, Mn), as revealed in Figure 3a. However, as shown in Figure 3b, the typical inclusions in 7MoSASS-Y were identified as YAlO_3_. Previous studies have shown that rare earth inclusions can serve as nucleation sites for austenite inhomogeneous transformation [25,29,38,39,40]. According to the two-dimensional disregistry calculation method provided by the literature [41], if the lattice disregistry is less than 12%, heterogeneous nucleation may occur, and when the lattice disregistry is below 6%, it is very favorable for heterogeneous nucleation, while the lattice disregistry value of YAlO_3_ with austenite (γ) is 2.9.

**Figure 4:**
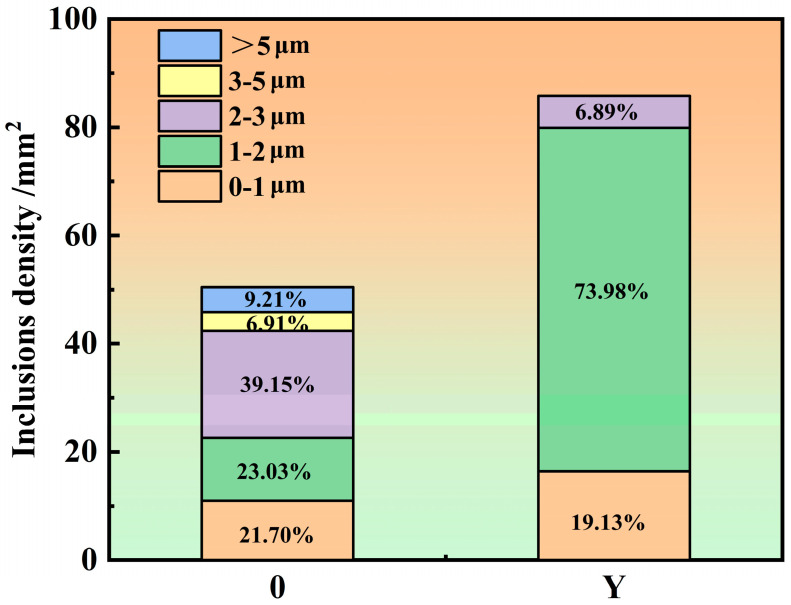
Inclusion of particle number density and the size distribution chart.

As depicted in Figure 4, the quantity density and size distribution of inclusions indicate that with the addition of Y, the quantity density of inclusions increased to 85.82/mm^2^. In addition, with the addition of Y, the proportion of inclusions with sizes between 1 and 2 μm increased to 73.98%. To sum up, Y increases the density of inclusions and decreases their size, and rare earth inclusions can act as heterogeneous nucleation cores for γ, thereby enhancing the heterogeneous nucleation ability of γ.

### 3.3. Effect of Y on the Solidification Kinetics of 7MoSASS

The nucleation process during solidification is mainly controlled by the interface, and the activation energy involved in this process is strongly influenced by catalytic factors. With the addition of rare earth elements, the nucleation mode also changes, and at the end of solidification, rare earth elements accumulate in the residual liquid phase, promoting the growth of free crystals in the undercooled melt. The Johnson–Mehl–Avrami–Kolmogorov (JMAK) theory, as shown in Formula (1), can be used to quantitatively analyze the nucleation and growth processes, thereby optimizing the material preparation process [29,42,43].

(1) $$Y=1-\exp(-k\cdot t^{n})$$

(2) $$k=k_{0}\cdot \exp(-\frac{Q}{RT})$$

**Figure 5:**
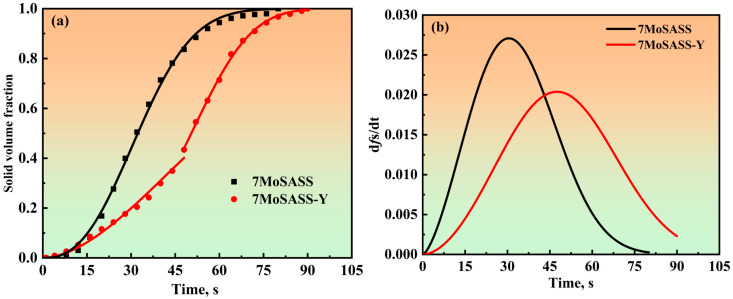
JMAK fitting graph. (**a**) fitted curves of the solid volume fraction as a function of time (**b**) Solidification growth rate graph.

The equation is commonly expressed as Equation (1), where *t* is the time, *k* is the rate constant, and *n* is the Avrami exponent. The rate constant *k* can be expressed as Equation (2), where *Q* is the activation energy, *k*_0_ is the dynamic parameter, *T* is the thermodynamic temperature, and *R* is the molar gas constant. When *n* is less than 3, the nucleation mode is saturated site nucleation, meaning nucleation occurs only at the phase transformation. When 3 < *n* < 4, the nucleation mode is a combination of saturated site nucleation and Avrami nucleation, where there is continued nucleation with a reduced nucleation rate after the initial nucleation occurs [44]. The fitting results are shown in Figure 5 and Table 4. Combining the in situ observations in Figure 2, it is evident that the addition of Y has altered the nucleation and growth mode of 7MoSASS. As shown in Figure 5a, the f(s)–t relationship for 7MoSASS follows the JMAK theory, indicating that at a cooling rate of 10 °C/min, the nucleation mode of 7MoSASS is saturated position nucleation. In contrast, for 7MoSASS-Y, a nucleation mode transition occurs when the solid fraction is around 0.5. As seen in Figure 5b, 7MoSASS primarily undergoes growth after nucleation, with austenite growing rapidly. In contrast, 7MoSASS-Y generates more nuclei in the early stages, with slower solidification growth rates. Moreover, new nuclei continue to form at a solid fraction of approximately 0.5. Therefore, we believe that Y has altered the nucleation and growth mode of 7MoSASS, shifting it from saturated site nucleation to a combination of saturated site nucleation + Avrami nucleation. The reasons behind this change will be further explored in the following discussion.

**Figure 6:**
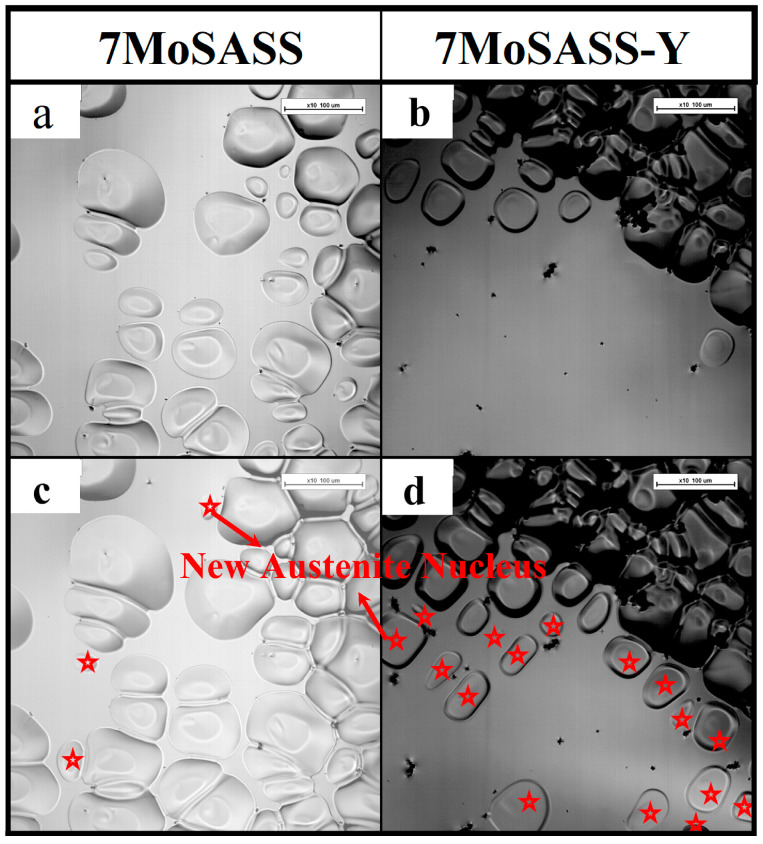
Real-time field images before and after the transition of nucleation mode: (**a**,**b**) before the inflection point; (**c**,**d**) after the inflection point.

**Figure 7:**
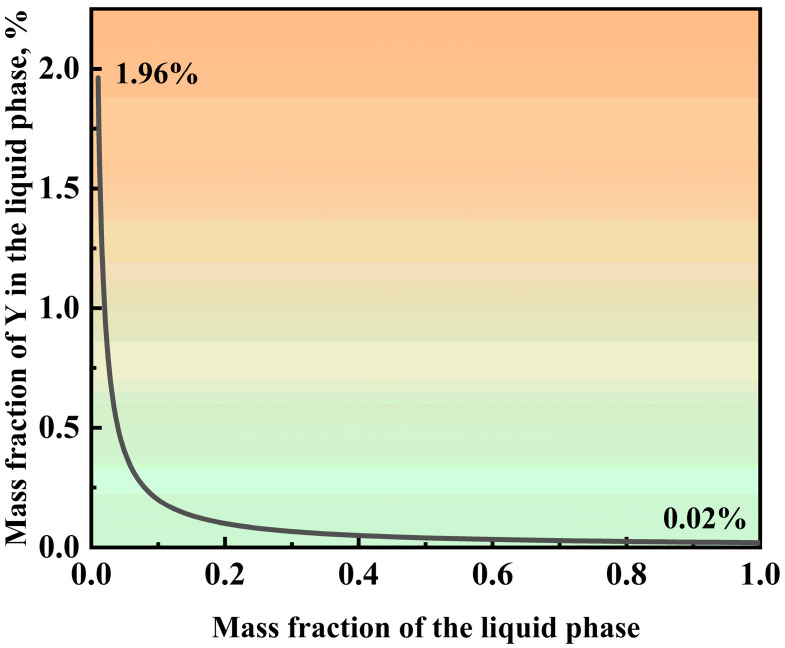
Mass fractions of Y in the liquid phase.

**Figure 8:**
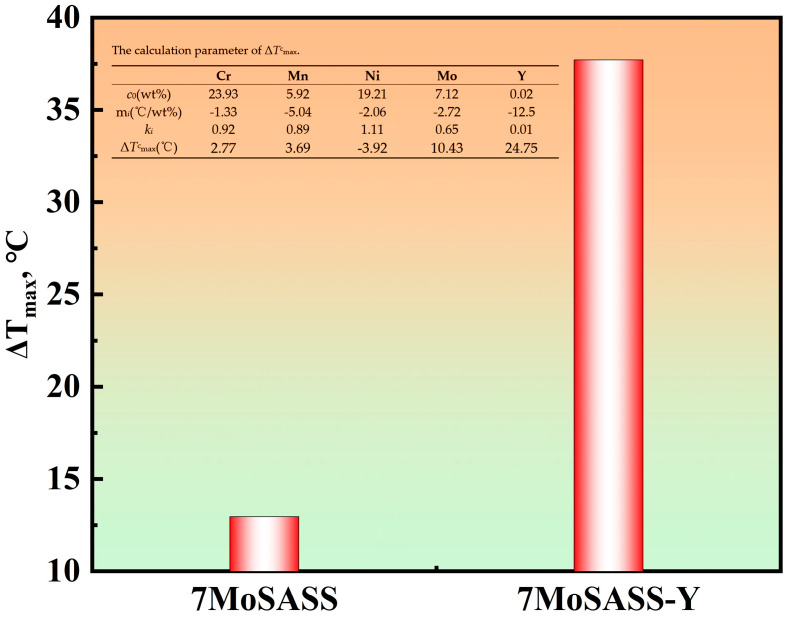
Δ*T*_max_ of 7MoSASS and 7MoSASS-Y.

Figure 6 displays real-time in situ images before and after the transition of nucleation modes. Figure 6c,d show real-time images of 7MoSASS and 7MoSASS-Y, respectively, revealing an increase in the number of nucleation sites by 34.48/mm^2^. This coincides with the JMAK kinetics analysis. As calculated through Thermo-Calc software (2021, Thermo-Calc, Stockholm, Sweden), the distribution of Y in the liquid phase is depicted in Figure 7. It can be observed that as the fraction of the liquid phase decreases, Y gradually accumulates within the liquid phase. This phenomenon aligns with the findings of Wang [29] and Zhang [25]. Y serves as a ferrite-forming element and exhibits low solubility in austenite. Moreover, it facilitates significant compositional undercooling, contributing to the refinement of the solidification microstructure of 7MoSASS. By applying Equation (3) [25], where $\Delta T_{\max}^{c}$ is the undercooling degree (°C) of element *i*; $c_{0}^{i}$ is the initial composition (wt.%) of element *i*; *m_i_* is the liquidus slope (°C/wt.%) of element *i*; and *k_i_* is the equilibrium partition coefficient of element *I*, the result is shown in Figure 8. Y can induce undercooling of 24.75 °C. As the nucleation mode shifts, Y exhibits a more favorable effect in grain refinement.

(3) $$\Delta T_{\max}^{c}=\frac{\sum c_{0}^{i}\times m_{i}\times (k_{i}-1)}{k_{i}}$$

### 3.4. Effect of Y on Element Segregation

**Figure 9:**
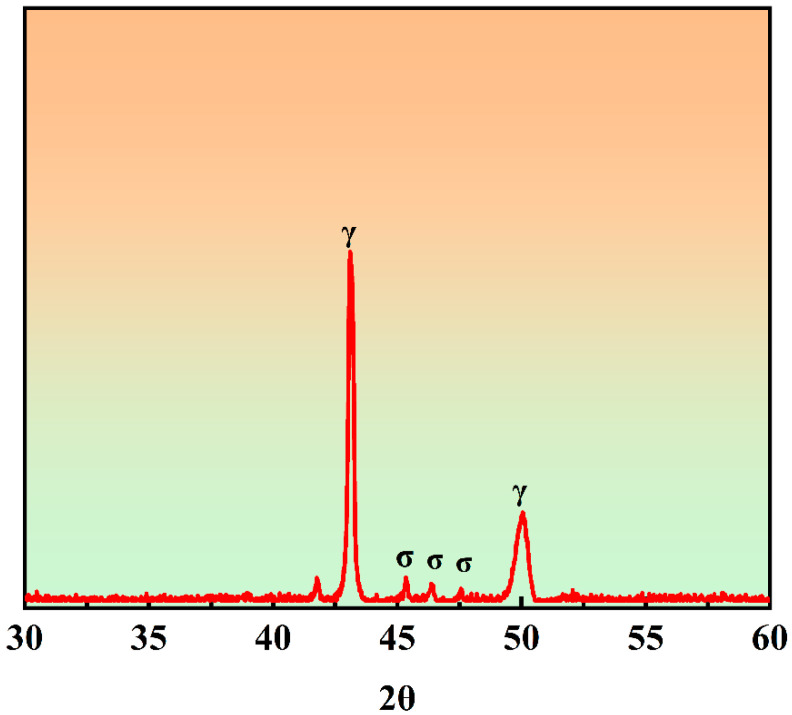
7MoSASS XRD.

**Figure 10:**
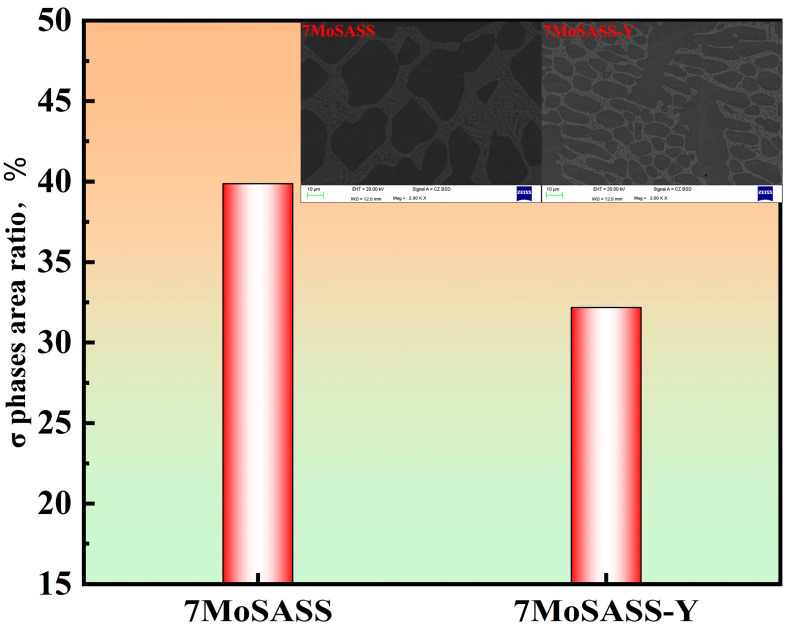
SEM-EDS analysis of the σ phase area statistics in 7MoSASS and 7MoSASS-Y samples.

Through XRD testing of the 7MoSASS (Figure 9), it was determined that the secondary phase present is the σ-phase. The SEM-EDS analysis results of the two sets of CLSM samples are shown in Figure 10. Firstly, from the morphological characteristics, it is evident that a significant number of secondary phases is present between dendrites, displaying features of eutectic decomposition. The addition of Y refines the solidification microstructure, and no large areas of precipitated phases were observed within the field of view. The precipitated phases take on a network-like structure, consistent with the in situ observation results. Further analysis of the σ-phase area fractions revealed that two samples exhibit relatively high proportions of the σ-phase, mainly influenced by the cooling rate. However, the addition of Y in the alloy resulted in a reduction in the σ-phase fractions by 7.5%.

**Figure 11:**
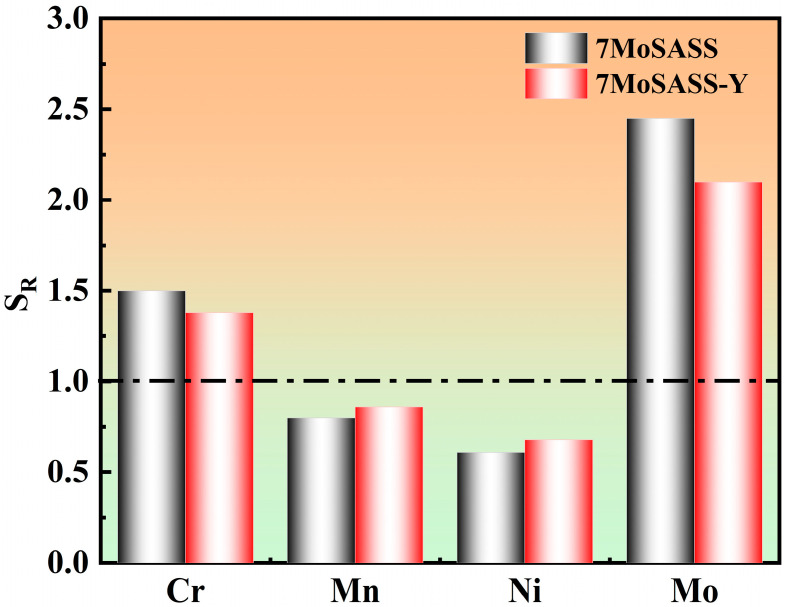
Element segregation ratios.

To further analyze the segregation behavior of the alloying elements in 7MoSASS, the segregation ratios (*SRs*) were calculated using Equation (4), which is as follows:

(4) $$SR=\frac{c_{interdendritic}^{\max}}{c_{dendritic}^{\min}}$$

where $c_{interdendritic}^{\max}$ represents the max concentration of the element in the interdendritic region and $c_{dendritic}^{\min}$ represents the min concentration of the element in the dendritic region. The results are illustrated in Figure 11 and showed that in 7MoSASS, Cr and Mo are the main elements undergoing segregation. The addition of Y effectively mitigates the segregation of Mo, with SR values decreasing by approximately 0.45. Notably, the SR_Mn_ is close to 1, indicating a more uniform distribution of Mn in the interdendritic regions. Mn plays a significant role in stabilizing the austenite phase, reducing the critical quenching rate of the steel and enhancing the stability of austenite during cooling, thus suppressing its decomposition. In summary, the addition of Y in the alloy has a positive effect on reducing the σ-phase fractions and mitigating the segregation of Mo, improving the overall stability and properties of the steel.

## 4. Conclusions

The effects of Y on the solidification process of 7MoSASS stainless steel were studied through in situ experiments and theoretical calculations. The main results are summarized as follows:

(1) Under low cooling rates (10 °C/min), the presence of Y can elevate the initial nucleation temperature and widen the solidification temperature range. Compared to 7MoSASS, the Y sample shows an increase of approximately 149.53/mm^2^ in austenite nucleation sites. The addition of Y is beneficial for improving the uniformity of the solidification microstructure.
(2) YAlO_3_ has a smaller lattice disregistry value with austenite, making it a suitable heterogeneous nucleation core for promoting the early nucleation of austenite. Moreover, during the final stages of solidification, Y tends to enrich to a greater extent, providing a higher degree of undercooling and promoting grain refinement in the solidification process.
(3) In 7MoSASS, the nucleation mechanism is primarily saturation site nucleation. However, with the addition of Y, a transition in the nucleation mechanism occurs at a solidification fraction of 50%, shifting from saturation site nucleation to a combination of saturation site nucleation + Avrami nucleation. The number of austenite nucleation sites in the Y sample exhibited increments of 34.48/mm^2^.
(4) The addition of Y resulted in a significant refinement of the solidification microstructure of 7MoSASS, with the proportion of precipitated phases decreasing by approximately 7.5% and exhibiting a reticular structure. Cr and Mo were the main elements exhibiting positive segregation in 7MoSASS.

## Figures and Tables

**Table 1:** Chemical composition of 7MoSASS (wt.%).

	C	Si	Mn	P	S	Cr	Ni	Mo	Cu	N	Al	Y
7MoSASS	0.01	0.03	5.92	0.004	0.004	23.93	19.21	7.12	0.43	0.48	0.006	--
7MoSASS-Y	0.01	0.03	6.13	0.006	0.003	23.96	19.17	7.16	0.43	0.47	0.008	0.016

**Table 2:** Analysis results of the solidification process.

10 °C/min	Tn (°C)	Grain Density/mm^2^		v = (g(10) − g(2))/8
		2 s	10 s	
7MoSASS	1367.2	4.30	21.52	2.15
7MoSASS-Y	1381.6	30.13	171.05	17.61

**Table 3:** Grain size statistics and variance analysis of the solidification structure.

	d_min_/μm	d_max_/μm	d_average_/μm	α
None	30.24	143.57	71.41	26.34
Y	27.18	106.39	65.07	20.01

**Table 4:** Fitting results obtained from the JMAK model.

	*k*	*n*	R^2^
7MoSASS	1.15 × 10^−4^	2.50 ± 0.07	0.99
7MoSASS-Y	5.13 × 10^−4^	1.78 ± 0.09	0.98
	5.91 × 10^−7^	3.56 ± 0.19	0.99

## Data Availability

Not applicable.
